# In vitro-generated alloantigen-specific Th9 cells mediate antileukemia cytotoxicity in the absence of graft-versus-host disease

**DOI:** 10.1038/s41375-020-0731-2

**Published:** 2020-02-07

**Authors:** Tanja Reisser, Daniel Halbgebauer, Jasmin Scheurer, Linda Wolf, Frank Leithäuser, Niklas Beyersdorf, Pamela Fischer-Posovszky, Klaus-Michael Debatin, Gudrun Strauss

**Affiliations:** 1 Department of Pediatrics and Adolescent Medicine, University Medical Center Ulm, Ulm, Germany; 2 Institute of Pathology, University of Ulm, Ulm, Germany; 3 Institute of Virology and Immunobiology, University of Würzburg, Würzburg, Germany

**Keywords:** Bone marrow transplantation, Cytokines

## To the Editor:

Allogeneic bone marrow transplantation (BMT) is a curative therapy for various hematological malignancies, such as leukemias and lymphomas. Donor-derived alloantigen-specific T cells eradicate residual tumor cells, however, these T cells are also responsible for the induction of graft-versus-host disease (GVHD). Therefore, one major challenge in BMT is the identification of T-cell subsets provoking antitumor cytotoxicity without causing GVHD. T helper (Th) subsets are programmed into various lineages dependent on ligand interactions and the cytokine milieu [1]. Th9 cells, which can be generated from naïve CD4 cells after activation in the presence of IL-4 and TGF-β are characterized by increased secretion of IL-9 and have been proven to exhibit efficient antitumor capacity especially toward melanomas [2–4]. Likewise, a few studies linked the presence of Th9/IL-9 with enhanced solid organ acceptance and decreased GVHD development [5–7]. However, the therapeutic effect of adoptive Th9 cell transfer on GVHD development and eradication of tumor cells is currently not well defined.

**Fig. 1:**
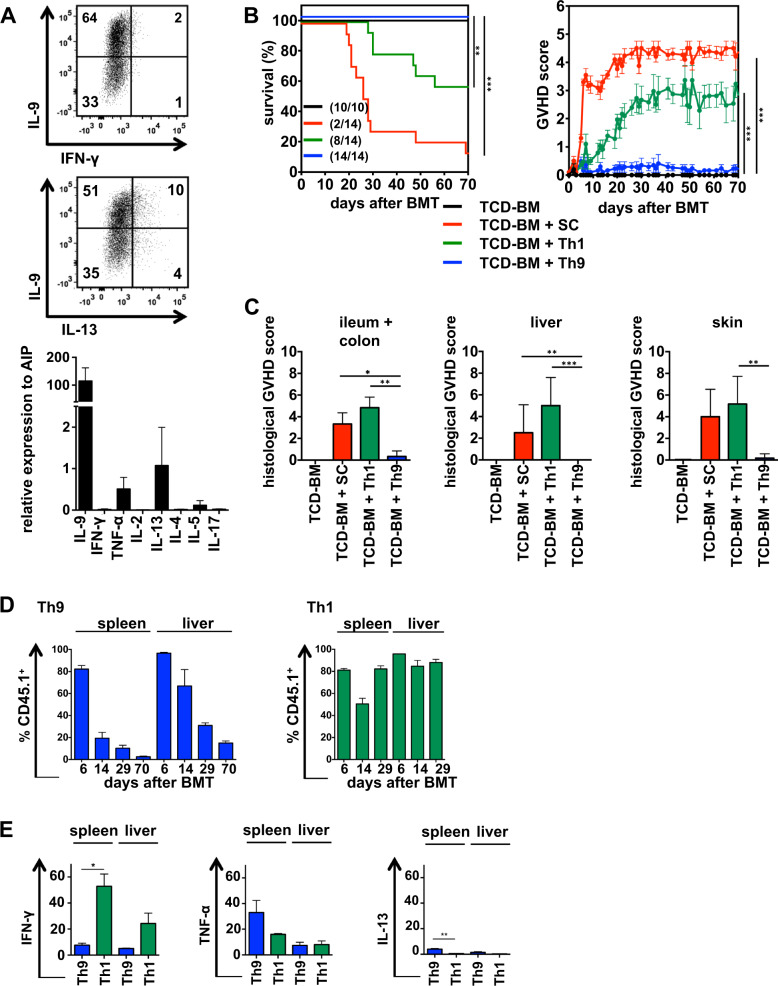
In vitro-generated Th9 cells do not induce GVHD in an allogeneic B6 → B6D2F1 BMT model but change their cytokine pattern in vivo. **a** Th9 cells were generated in vitro from naïve CD4^+^ T cells of B6 (H-2^b^) mice. On day 5, cells were stained intracellularly for IL-9, IFN-γ and IL-13 expression or were analyzed by qRT-PCR for cytokine expression. **b**–**e** Th9 and Th1 cells were generated in vitro from B6.SJL (H-2^b^, CD45.1) mice. Lethally irradiated B6D2F1 (H-2^bxd^, CD45.2) recipient mice were reconstituted with TCD-BM from B6 (H-2^b^, CD45.2) mice alone or together with spleen cells (SC), Th9 or Th1 cells. **b** Survival and GVHD scores were determined. Surviving animals/total animals treated are indicated in brackets. Error bars of GVHD score indicate mean ± SEM. **c** Paraffin sections of GVHD target tissues were analyzed for histological signs of GVHD on the day mice were euthanized due to their moribund state or at the end of the experiment. Different days after BMT, splenocytes and liver lymphocytes were stained for CD45.1 to define homing and survival of transplanted Th1 and Th9 cells (**d**) and analyzed for their intracellular cytokine expression day 29 after BMT (**e**). **a** Representative FACS staining from one out of more than 10 independent experiments done; *N* = 3 independent qRT-PCRs, data are shown as mean ± SD. **b** Survival: TCD-BM + Th1 vs TCD-BM + Th9, **P ≤ 0.01 and TCD-BM + SC vs TCD-BM + Th9, ***P ≤ 0.001; GVHD Score: paired Student’s *t* test: TCD-BM + Th1 vs TCD-BM + Th9 and TCD-BM + SC vs TCD-BM + Th9, ***P ≤ 0.001. Data represent two independent experiments. **c** Data represent a single experiment with *N* = 5 mice /group; paired Student’s *t* test: *P ≤ 0.05; **P ≤ 0.01, ***P ≤ 0.001. **d**, **e**. Data represent the mean value ± SD of three mice/group on each day and are representative for two independent experiments performed. paired Student’s *t* test: *P ≤ 0.05; **P ≤ 0.01.

To clarify whether Th9 cells are suitable for donor lymphocyte infusion in BMT settings, we differentiated naïve splenic CD4^+^ T cells derived from B6 mice on anti-CD3/CD28 coated plates in the presence of IL-4, TGF-β and the TNF-family cytokine TL1A. About 60% of the cells expressed IL-9 intracellulary after 5 days of differentiation and exhibited high IL-9 RNA expression. Expression of the Th1-specific cytokine IFN-γ was undetectable while IL-5, -13 and TNF-α were weakly expressed (Fig. 1a). Most importantly, Tregs were not differentiated, because Foxp3 expression was undetectable (Fig. S1). To clarify the impact of Th9 cells on GVHD induction, we used an allogeneic CD4^+^-dependent parent → F1 BMT model with a 50% mismatch for MHC class I and II molecules (B6 → B6D2F1). The alloantigen H-2^d^ expressed on recipient tissue of lethally irradiated B6D2F1 (H-2^bxd^) mice activates transplanted B6-derived (H-2^b^) donor T cells. Acute, lethal GVHD in this model only develops if the transplant contains mature CD4^+^ T cells since the transfer of CD8^+^ T cells alone induces only moderate histological GVHD [8]. B6D2F1 mice were reconstituted with B6-derived TCD-BM alone or in combination with total spleen cells (SC) or Th9 cells. To exclude that Th9 functionality is impaired by the in vitro activation and differentiation process, we also transferred in vitro-generated Th1 cells, which are characterized by their IFN-γ and TNFα expression (Fig. S2) and are known to be main drivers of GVHD. Th9 and Th1 cells established from B6.SJL mice express the congenic marker CD45.1 allowing the dissection of transplanted in vitro-generated Th subsets (CD45.1^+^) from T cells of host origin (B6D2F1; CD45.2^+^) or T cells derived from transplanted hematopoietic stem cells (B6; CD45.2^+^). While allogeneic spleen cells (SC) and in vitro-generated Th1 cells induced severe clinical and histological GVHD associated with high GVHD scores, mice treated with Th9 cells showed no signs of GVHD (Fig. 1b, c). Missing GVHD-inducing capacity of Th9 cells was confirmed by low levels of GVHD-associated cytokines IFN-γ and TNF-α in the serum of transplanted mice (Fig. S3). Likewise, Th9 transfer in a second allogeneic CD4^+^-dependent BMT model (B6 → B6.bm12) did not induce GVHD (Fig. S4). In vivo expansion and homing of Th9 cells were detectable in liver and spleen until 70 days after BMT, however, Th9 cells decreased over time more rapidly than transferred Th1 cells (Fig. 1d). Most interestingly, Th9 cells changed their cytokine expression profile in vivo. 29 days after BMT transplanted Th9 cells in spleen and liver produced IFN-γ, TNF-α and IL-13 but no IL-9, while Th1 cells maintain their cytokine pattern (Fig. 1e).

**Fig. 2:**
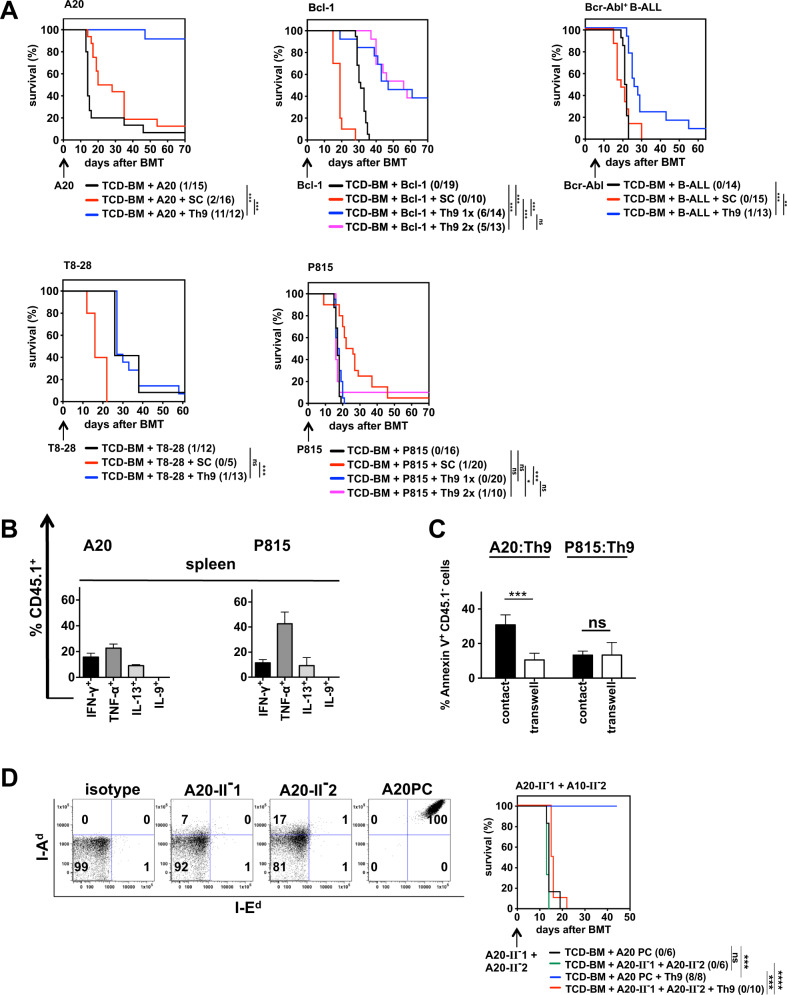
In vitro-generated Th9 cells mediate the GVT effect selectively toward B-cell malignancies. **a** Th9 cells were generated in vitro from B6 (H-2^b^) mice. Lethally irradiated B6D2F1 (H-2^bxd^, CD45.2) recipient mice were reconstituted with TCD-BM from B6 (H-2^b^, CD45.2) mice in the presence or absence of B6-derived (H-2^b^) SCs or Th9 cells. Th9 cells were given only once at the day of BMT (1×) or twice at the day of BMT and 6 days later (2×). Tumor cells were injected either on the day of BMT (A20 (H-2^d^), Bcl-1 (H-2^d^), T8-28 (H-2^d^), P815 (H-2^d^) or seven days later (Bcr-Abl^+^ B-ALL (H-2^d^, GFP^+^)) as indicated by arrows. Surviving animals/total animals treated are indicated in brackets. **b** Th9 cells were generated in vitro from B6.SJL (H-2^b^, CD45.1) mice and co-injected into lethally irradiated B6D2F1 (H-2^bxd^, CD45.2) recipient mice reconstituted with B6-derived TCD-BM (H-2^b^, CD45.2). At the day of BMT, mice were injected with B-cell lymphoma A20 (H-2^d^) or mastocytoma P815 (H-2^d^). 14 days after BMT, splenocytes and liver lymphocyte were stained for CD45.1^+^ Th9 cells, which were intracellulary stained for IFN-γ, TNF-α, IL-13 and IL-9 expression. **c** B6.SJL-derived in vitro-generated Th9 cells were co-cultured in direct cell-to-cell contact or in transwells with A20 (H-2^d^, CD45.2) or P815 (H-2^d^, CD45.2). After 48 h, Annexin V expression was determined on CD45.1^−^ tumor cells. **d** Downregulation of MHC class II by CRISP/Cas9-mediated targeting of CIITA in A20 cells was confirmed by flow cytometry. Two MHC class II downregulated A20 cell lines (A20-II^-^1 and A20-II^-^2, GFP^+^) and the control A20 cells transfected with nontargeting sgRNA (A20PC, H-2^d^, GFP^+^) were stained for I-E^d^ and I-A^d^ or isotype control. Lethally irradiated B6D2F1 mice were reconstituted with B6-derived TCD-BM in the absence of presence of in vitro-generated B6.SJL-derived Th9 cells. Mice were injected at the day of BMT with a 1:1 mixture A20-II^-^1 and A20-II^-^2 or the MHC Cl II expressing control A20PC. Surviving animals/total animals treated are indicated in brackets. **a** *P ≤ 0.0, **P ≤ 0.01, ***P ≤ 0.001; ns not significant. Data represent 1 (Bcr-Abl), 2 (Bcl-1, T8-28) or three independent experiments (A20, P815) for each tumor cell line. **b** Data represent the mean value ± SD of three mice/group. **c** Data represent the mean value ± SD of triplicates of two independent experiments with ***P ≤ 0.001; ns not significant, paired Student’s *t* test. **d** Representative FACS staining from more than five stainings performed. Data represent a single experiment with indicated mice numbers. ***P ≤ 0.001, ****P ≤ 0.0001; ns not significant.

Since Th9 cells efficiently eradicate solid tumors, we next defined their capacity to destroy hematological tumors in the context of allogeneic BMT. First, we co-transplanted three H-2^d^ expressing B-cell tumors, the lymphoblast cell lines A20 and Bcl-1 and a GFP-expressing Bcr-Abl-transformed B-acute lymphoblastic leukemia resembling more closely primary leukemic cells (Fig. 2a). In all tumor models used, mice developed lethal tumors if only BM cells were transplanted, while the co-transplantation of spleen cells eradicated tumor cells but induced GVHD. Adoptive transfer of Th9 cells into A20-bearing animals led to complete tumor eradication in more than 92% of the mice. At day 70 the BM of Th9-transplanted mice was free of tumor cells (Fig. S5A). Bcl-1 injection led to tumor-induced death in 100% of mice reconstituted with BM cells alone. A single injection of Th9 cells at the day of BMT (day 0, 1×) rescued 43% of the treated mice, which could not be improved (39%) by two-time Th9 injection (day 0 and day 6, 2×). However, repeated Th9 treatment led to improved Bcl-1 eradication (H2-K^d^-positive cells) since residual tumor cells were undetectable in the spleen at the end of experiment (Fig. S5B). Since Bcr-Abl^+^B-ALL cells killed the animals in less than 14 days (data not shown), B-ALL cells were injected 7 days after transplantation to prevent tumor-mediated death before reconstitution of the hematopoietic compartment. Th9-treated mice exhibited a significant prolonged survival compared to animals receiving BM alone, however, only one mouse survived until the end of the experiment and was tumor free (GFP^neg^) (Fig. S5C). Due to B-cell eradication by Th9 cells, we defined the GVT effect toward mastocytoma cells (P815, H-2^d^) or T-cell lymphoma cells (T8-28, H-2^d^). Most interestingly, Th9 cells were totally ineffective in preventing growth of both tumors (Fig. 2a, Fig. S5D, E). Even two consecutive Th9 injections at day 0 and 7 after BMT (2×) in P815-treated mice showed no distinct effect. Only a single mouse survived, which, however, was tumor free clearly indicating that cellular therapy with adoptively transferred Th9 cells is only applicable in eliminating B-cell tumors.

Since IL-9 is a main driver of the antitumor effect against melanomas, the contribution of IL-9 for the selective GVT effect was defined. However, independent whether Th9-transplanted mice received A20 or P815 cells no IL-9 production of re-isolated Th9 was detectable 14 days after BMT. However, transplanted Th9 cells produced IFN-γ, TNF-α and IL-13 (Fig. 2b). To define the role of soluble factors such as IFN-γ and TNF-α in tumor eradication, we performed transwell experiments by co-culturing Th9 cells with A20 or P815 cells. However, only direct cell-to-cell contact-induced apoptosis in A20 cells, while P815 cells were not attacked at all (Fig. 2c). One unique feature of B cells tumors compared to most other hematological tumors is the constitutive expression of MHC class II molecules, which can transduce apoptosis signals [9]. Therefore, we abrogated MHC class II expression using CRISP/Cas9-mediated targeting of the MHC class II transactivator (CIITA), which is responsible for class II expression [10]. Using two different sgRNAs for CIITA in two independent transfections resulted in two A20 cell lines (A20-II^-^1, A20-II^-^2) with complete absence of I-E^d^ expression and strongly downregulated I-A^d^ expression, whereas A20 cells transfected with nontargeting sgRNAs (A20PC) exhibited high expression of both molecules. In BM-transplanted mice the injection of a 1:1 mixture of both Class II^−/−^ A20 cells induced tumor growth in 100% of the mice comparable to A20PC cells expressing MHC class II. Most importantly, class II deficiency rendered them insensitive for eradication by adoptively transferred Th9 cells. All mice died due to tumor development, while all mice, which received the class II expressing A20PC cells survived (Fig. 2d, Fig. S6) impressively showing that MHC class II confers Th9 sensitivity.

To our knowledge, we show for the first time that the adoptive transfer of Th9 cells exhibit an efficient GVT effect selectively toward B-cell tumors in the absence of GVHD induction. Th9 functions were primarily defined as proinflammatory, but over time the potent antitumor activity of Th9 cells was discovered [2, 4]. However, antitumor effects refer to eradication of solid tumors, while the role of Th9 /IL-9 toward hematopoietic tumors is unclear. As IL-9 is the driving force in melanoma eradication, the action of IL-9 on hematopoietic cells appears to be rather tumor promoting. IL-9 overexpressing mice develop thymomas [11] and IL-9 promotes disease pathogenesis in patients with hematological cancers [12, 13]. The discrepancy to our findings might be explainable by the change of cytokine expression in Th9 cells after adoptive transfer. IL-9 is not further produced and a shift toward IFN-γ and TNF-α secretion occurred. Although these cytokines have antitumor features, the requirements of direct cell-to-cell contact for tumor destruction exclude a key role of IFN-γ and TNF-α in tumor eradication. Instead, the expression of MHC class II molecules sensitizes B cells for Th9-mediated killing and explains why MHC class II negative hematological tumors are protected from Th9-mediated destruction. MHC class II expression on B cells might be required for continuous activation of Th9 cells in vivo or most likely transmits apoptosis-inducing signals after activation by Th9 cells since class II-mediated apoptosis was reported in different cell types [9, 14, 15]. In summary, in vitro-differentiated Th9 cells represent a Th subset selectively eradicating B-cell malignancies in experimental models of BMT in the absence of GVHD induction, which might define Th9 cells as an attractive candidate for donor lymphocyte infusions to improve long-term tumor-free survival.

## Supplementary information

Supplement
