# Supplementary material for: In vitro-generated alloantigen-specific Th9 cells mediate antileukemia cytotoxicity in the absence of graft-versus-host disease
Source: Leukemia. 2020 Feb 7;34(7):1943–8. doi: 10.1038/s41375-020-0731-2 (PMC7326704; doi:10.1038/s41375-020-0731-2)
Supplement: Supplementary file 1 — Supplement [file 41375_2020_731_MOESM1_ESM.pdf]

Supplement: Material and Methods, Tables, Figures

Materials and Methods

Mice and BMT

Female C57BL/6 (B6; H-2^b, CD45.2), B6D2F1 (H-2^{bxd}, CD45.2) (Janvier, France), B6.C-H2-bm12 (B6.bm12; H2-$Ab1^{bm12}$, CD45.2), B6.SJL-$Ptprc^a Pepc^b$/BoyJ (B6.SJL; H-2^b, CD45.1) mice (breeding pairs from The Jackson Laboratory and bred at Ulm University, Germany) were used (6 to 12 weeks of age). Bone marrow transplantation (BMT) including T cell depletion (TCD) of BM was done according to Messmann *et al.*[1]. 5 x 10^6 TCD-BM was co-injected with 2 x 10^7 splenocytes (SC) or 5 x 10^6 in vitro-generated Th9 or Th1 cells. GVHD scores were determined by analyzing weight, activity, skin, fur ruffling, and posture according to Cook *et al.*[2]. Animals euthanized due to their moribund state during the experiment remained included in the calculation until the end of experiment with their final GVHD scores. A20 (H-2^d, 1x10^6 cells/mouse), A20PC (H-2^d, 1x10^6 cells/mouse), A20-II^-1+A20-II^-2 (H-2^d, 5x10^5+5x10^5 cells/mouse), Bcl-1 (H-2^d, 3x10^3 cells/mouse), T8-28 (H-2^d, 3x10^3 cells/mouse) or P815 (H-2^d, 2x10^3 cells/mouse) were iv injected at the day of BMT while Bcr-Abl^+-B-ALL (H-2^d, GFP^+, 3x10^2 cells/mouse) were iv injected 7 days after BMT. All animal experiments were performed according to the international regulations for the care and use of laboratory animals and were approved by the local Ethical Committee Regierungspräsidium Tübingen, Germany.

Cell culture

Tumor cell lines were grown in RPMI 1640 (Thermo Fisher), 10% fetal calf serum (FBS; Sigma-Aldrich), 2 mM L-glutamine, 1 mM sodium pyruvat, 50 µM β-mercaptoethanol, 100 U/ml Penicillin and 100 µg/ml Streptomycin at 37 °C and 7,5% CO_2. Cell lines were originally obtained from ATCC or provided by cooperation partners. Bcl-1 and T8-28: N.

Beyersdorf[3], Bcr-Abl$^+$-B-ALL: J. Duyster[4]. Cells were regularly cultured in Mycoplasma Removal Agent and checked for surface marker expression every 2 month. Transwell experiments were performed with Thin Cert Tissue Culture Inserts (pore size 0.4 µm) in 12 well-plates (Greiner bio-one) in a ratio of tumor cells to in vitro-generated Th9 cells of 1 : 10.

T cell differentiation

Naive CD4$^+$ T cells were purified from spleen and lymph nodes via Naive CD4$^+$ T cell Isolation Kit (Miltenyi Biotech). T cell subsets were differentiated according to table S1 for 3 days at 37 °C and 7,5% CO_2 followed by expansion with fresh growth media in the presence of the appropriate cytokines at the same concentration as on day 0 until day 5.

Organ preparation and histopathology

Single cell suspension of spleen was prepared by gently pressing the spleen through a cell strainer (pore size 70 µm) followed by lysis of erythrocytes. Isolation of the liver lymphocyte fraction and histopathology was done according to Messmann *et al.*[1] and Kaplan *et al.*[5].

Flow cytometry

A total of 5 x 10^5 cells were stained with antibodies listed in table S2 and measured on LSR II flow cytometer (BD Bioscience). Dead cells were defined by 7-amino-actinomycin-D (7-AAD, Sigma-Aldrich), FSC/SSC or by Annexin V staining (Annexin-V-Fluos Staining Kit, Roche Diagnostics). For intracellular cytokine detection, cells were restimulated with phorbol myristate acetate (PMA) (20 ng/ml), ionomycin (1 µM) (Calbiochem) and brefeldin A (10 µg/ml) (Sigma-Aldrich) for 5 hours. Cells were stained for CD3, CD4, CD8 or CD45.1, fixed with 4% paraformaldehyde (PFA), lysed with 0.1% saponin (Sigma-Aldrich) and stained for cytokines. Foxp3 expression was stained with Foxp3 Transcription Factor Staining Buffer Kit (Thermo Fisher).

qRT-PCR

RNA was isolated and complementary DNA was synthesized as previously described[6] and qRT-PCR was performed with a CFX Connect™ Real-Time PCR Detection System (Bio-Rad) using a LightCyler FastStart DNA Master PLUS SYBR Green I Kit (Roche Diagnostics). qRT-PCR results were normalized using mouse aryl hydrocarbon receptor-interacting protein (AIP) as house keeping gene. Primer (Biomers) used are listed in table S3.

Serum cytokines

Serum cytokines of blood collected after BMT were analyzed with ProcartaPlex Multiplex Immunoassay (Thermo Fisher).

CRISP/Cas9-mediated CIITA targeting

CRISPR/Cas9-mediated knockout of CIITA was performed to generate MHC class II-deficient cells. Two SgRNA duplexes targeting the CIITA gene (CIITA (1) sgRNA-top: 5'-CCC GGA GCC TTA GTC GAG CT-3'; CIITA (1) sgRNA-bottom: 5'- AG CTC GAC TAA GGC TCC GGG-3'; CIITA (2) sgRNA-top: 5'-GAG CGC CAG CTA GCC CAC GG-3'; CIITA (2) sgRNA-bottom: 5'- CC GTG GGC TAG CTG GCG CTC-3') and a non-targeting control (non-targeting sgRNA-top: 5'-GGT CAC CGA TCG AGA GCT AG-3'; non-targeting sgRNA-bottom: 5'-GA TGG CGC TTC AGT CGT CGG-3') were cloned into the pMuLE ENTR U6 stuffer sgRNA scaffold L1-R5 plasmid (Multiple Lentiviral Expression Kit, Addgene #1000000060, kindly provided by Ian Frew)[7]. Correct insertion of the sgRNA duplexes was verified by Sanger sequencing. Using LR Clonase II Plus (ThermoFisher Scientific), the specific pMuLE ENTR U6-sgRNA and pMuLE ENTR CMV-hCas9 L5-L2 plasmids were recombined with a SleepingBeauty transposon plasmid 'pMuSE eGFP-P2A-PuroR DEST' that had been generated earlier by our laboratory[8].

Murine A20 cells were co-transfected with the resulting pMuSE U6-sgRNA + CMV-hCas9 + RPBSA-eGFP-P2A-PuroR plasmids and the SleepingBeauty-expressing pCMV(CAT)T7-SB100 plasmid (Addgene #34879, kindly provided by Zsuzsanna Izsvak) at a mass ratio of 19:1 using a Neon Transfection System (ThermoFisher Scientific) with 2 × 10 ms pulses of 1400 V. Stable cultures were generated by sorting for GFP and down-regulated I-A^d and I-E^d. MHC class II deficiency was confirmed by FACS using I-A and I-E antibodies.

Statistics

To ensure robust and unbiased results, experiments have been repeated several times or were performed with sufficient animal numbers. Comparison between two groups with similar variance were analyzed using paired Student's t-test. Kaplan–Meier method and Log-Rank was used for statistics of survival. Statistical tests were performed with GraphPad Prism 6. Results were considered significant if $P \leq 0.05$. Data represent mean±SD with the exception of GVHD scores showing mean±SEM. Animal numbers were chosen according to the statistical report created by a statistician to obtain a significance level of 0.05 and a power level of 0.80. Randomization of animals were not necessary since mice used for transplantation were delivered at defined ages and sexes and exhibited no interindividual differences. The investigator was not blinded to the groups.

Table S1. Differentiation conditions for Th9, Th1 and Treg cells.

**Th9 cells (1 x10^6/ml)**	**reagent**	**concentration**	**manufacturer**
plate-bound co-stimulation	Purified Hamster Anti-Mouse CD3e (clone 145-2C11)	1 µg/ml	BD
	Purified NA/LE Hamster Anti-Mouse CD28 (clone 37.51)	1 µg/ml	BD
cytokines	Recombinant human TGF-β	5 ng/ml	Peprotech
	Recombinant murine IL-4	10 ng/ml	Peprotech
	Recombinant mouse TL1A/TNFSF15	10 ng/ml	R&D
	InVivoMab anti-mouse IFNgamma (clone XMG1.2)	10 ug/ml	Hölzel Diagnostika
medium	IMDM		Thermo Fisher
medium additives	FBS	5%	Sigma-Aldrich
	L-Glutamin	2 mM	Thermo Fisher
	Sodium-Pyruvat	1 mM	Thermo Fisher
	β-mercaptoethanol	50 µM	Thermo Fisher
	Penicillin	100 U/ml	Thermo Fisher
	Streptomycin	100 µ/ml	Thermo Fisher
**Th1 cells (0,5 x10^6/ml)**	**reagent**	**concentration**	**manufacturer**
plate-bound co-stimulation	Purified Hamster Anti-Mouse CD3e (clone 145-2C11)	2,5 µg/ml	BD
	Purified NA/LE Hamster Anti-Mouse CD28 (clone 37.51)	3 µg/ml	BD
cytokines	Recombinant murine IL-2	5 ng/ml	Peprotech
	Recombinant murine IL-12	20 ng/ml	Peprotech
	InVivoMab anti-mouse IL-4 (clone 11B11)	10 ug/ml	Hölzel Diagnostika
medium	RPMI 1640		Thermo Fisher
medium additives	FBS	10%	Sigma-Aldrich
	L-Glutamin	2 mM	Thermo Fisher
	Sodium-Pyruvat	1 mM	Thermo Fisher
	β-mercaptoethanol	50 µM	Thermo Fisher
	Penicillin	100 U/ml	Thermo Fisher
	Streptomycin	100 µ/ml	Thermo Fisher
**Treg cells (1 x10^6/ml)**	**reagent**	**concentration**	**manufacturer**
plate-bound co-stimulation	Purified Hamster Anti-Mouse CD3e (clone 145-2C11)	3 µg/ml	BD
	Purified NA/LE Hamster Anti-Mouse CD28, (clone 37.51), add soluble	3 µg/ml	BD
cytokines	Recombinant murine IL-2	5 ng/ml	Peprotech
	Recombinant human TGF-β	5 ng/ml	Peprotech
medium	RPMI 1640		Thermo Fisher
medium additives	FBS	10%	Sigma-Aldrich
	L-Glutamin	2 mM	Thermo Fisher
	Sodium-Pyruvat	1 mM	Thermo Fisher
	β-mercaptoethanol	50 µM	Thermo Fisher
	Penicillin	100 U/ml	Thermo Fisher
	Streptomycin	100 µ/ml	Thermo Fisher

Table S2. Antibodies for Flow Cytometry.

Epitope	clone	fluorochrom	manufacturer
CD3	17A2	AF700, APC	Thermo Fisher
CD3	145-2C11	PeCy7	Thermo Fisher
CD4	RM4-5	APCef780	Thermo Fisher
CD8	53-6.7	PeCy7, APC, PB	Thermo Fisher
CD19	1D3	PE, PerCP-Cy5.5	BD
CD45.1	A20	V450, FITC	BD, Thermo Fisher
H-2Kb	AF6-88.5.5.3	APC	Thermo Fisher
H-2Kd	SF1-1.1.1	ef450	Thermo Fisher
IL-9	RM9A4	APC	BioLegend
IFN-γ	XMG1.2	PE	Thermo Fisher
TNF-α	MP6-XT22	PE	Thermo Fisher
IL-13	eBio13A	PE	Thermo Fisher
Foxp3	FJK-16s	AF700, APC	Thermo Fisher
I-E^d	14-4-4S	PE	Thermo Fisher
I-A^d	AMS-32.1	APC	Thermo Fisher
mIgG2a	eBMG2a	PE	Thermo Fisher
mIgG2b	eBMG2b	APC	Thermo Fisher

Table S3. Primer pairs for qRT-PCR.

IL-2 forward	AACCTGAAACTCCCCAGGAT
IL-2 reverse	GTCAAATCCCAGAACATGCCG
IL-9 forward	AACAGTCCCTCCCTGTAGCA
IL-9 reverse	AAGGATGATCCACCGTCAAA
IL-4 forward	GGTGTTCTTCGTTGCTGTGA
IL-4 reverse	TCTCGAATGTACCAGGAGCC
IL-5 forward	CCCACGGACAGTTTGATTCT
IL-5 reverse	GCAATGAGACGATGAGGCTT
IL-13 forward	CACACTCCATACCATGCTGC
IL-13 reverse	TGTGTCTCTCCCTCTGACCC
IL-17 forward	TGAGCTTCCCAGATCACAGA
IL-17 reverse	TCCAGAAGGCCCTCAGACTA
TNFα forward	CCAGACCCTCACACTCAGATCATCTTCTC
TNFα reverse	CTAGTTGGTTGTCTTTGAGATCCATGCCGT
IFN-γ forward	TGCAGAGCCAGATTATCTCTTTCTACCTCAG
IFN-γ reverse	GGTTGTTGACCTCAAACTTGGCAATACTC
AIP forward	GCTCCGTTATAGATGACAGC
AIP reverse	ATCTCGATGTGGAAGATGAG

1. Messmann JJ, Reisser T, Leithauser F, Lutz MB, Debatin KM, Strauss G. In vitro-generated MDSCs prevent murine GVHD by inducing type 2 T cells without disabling antitumor cytotoxicity. Blood 2015; 126: 1138-1148.

2. Cooke KR, Kobzik L, Martin TR, Brewer J, Delmonte J, Jr., Crawford JM*, et al.* An experimental model of idiopathic pneumonia syndrome after bone marrow transplantation: I. The roles of minor H antigens and endotoxin. Blood 1996; 88: 3230-3239.

3. Beyersdorf N, Werner S, Wolf N, Herrmann T, Kerkau T. Characterization of a new mouse model for peripheral T cell lymphoma in humans. PLoS One 2011; 6: e28546.

4. Miething C, Grundler R, Mugler C, Brero S, Hoepfl J, Geigl J*, et al.* Retroviral insertional mutagenesis identifies RUNX genes involved in chronic myeloid leukemia disease persistence under imatinib treatment. Proc Natl Acad Sci U S A 2007; 104: 4594-4599.

5. Kaplan DH, Anderson BE, McNiff JM, Jain D, Shlomchik MJ, Shlomchik WD. Target antigens determine graft-versus-host disease phenotype. J Immunol 2004; 173: 5467-5475.

6. Husecken Y, Muche S, Kustermann M, Klingspor M, Palmer A, Braumuller S*, et al.* MDSCs are induced after experimental blunt chest trauma and subsequently alter antigen-specific T cell responses. Sci Rep 2017; 7: 12808.

7. Albers J, Danzer C, Rechsteiner M, Lehmann H, Brandt LP, Hejhal T*, et al.* A versatile modular vector system for rapid combinatorial mammalian genetics. J Clin Invest 2015; 125: 1603-1619.

8. Tews D, Pula T, Funcke JB, Jastroch M, Keuper M, Debatin KM*, et al.* Elevated UCP1 levels are sufficient to improve glucose uptake in human white adipocytes. Redox Biol 2019; 26: 101286.

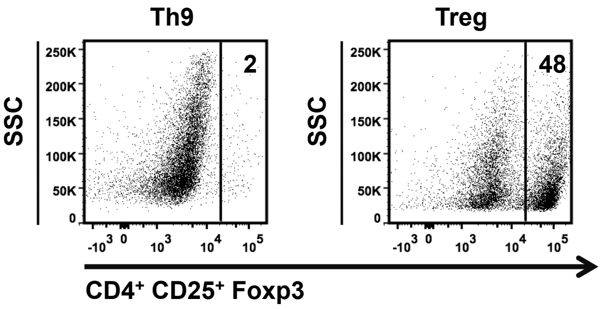

Figure S1. In vitro-generated Th9 cells do not express Foxp3.
Th9 and Tregs were generated from B6 mice and stained for CD4 and CD25 and $CD4^+CD25^+$ cells were analyzed for Foxp3 expression (Foxp3/Transcription Factor Staining Buffer Set, Thermo Fisher) by flow cytometry.

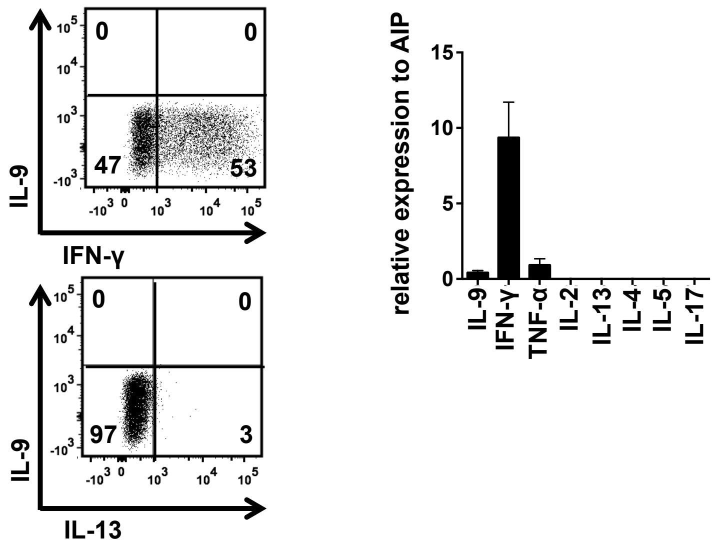

Figure S2. Characterization of in vitro-generated Th1 cells.
Th1 cells were generated from B6 mice and stained for cytokines intracellularly (representative FACS staining from N = 5 independent experiments). Cytokine expression was also analyzed by qRT-PCR (N = 3 independent experiments, data are shown as mean ± SD).

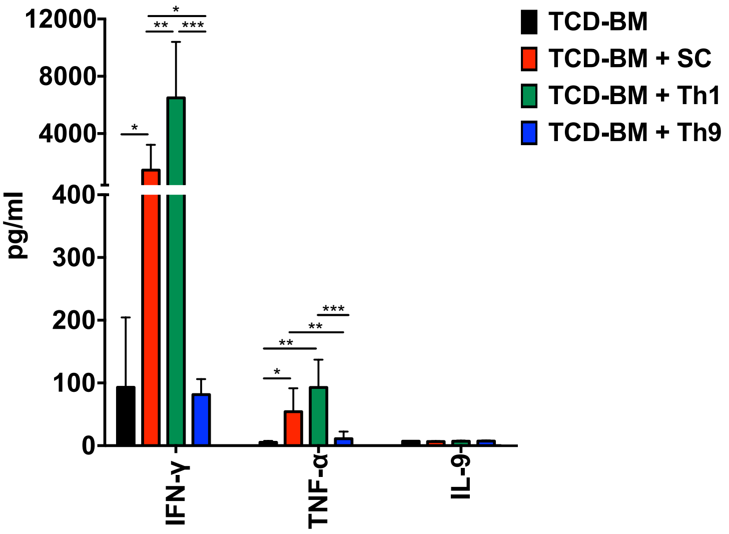

Figure S3. GVHD-associated cytokines are expressed at very low levels in the serum of Th9-transplanted mice.
Nine days after BMT, serum from mice reconstituted with TCD-BM and spleen cells (SC), Th1 or Th9 cells was analyzed with ProcartaPlex Multiplex Immunoassay (Thermo Fisher). (N = 5 (TCD-BM), 10 (TCD-BM+SC), 14 (TCD-BM+ Th1/Th2), paired *t* test, data are shown as mean ± SD; *P ≤ 0.05; **P ≤ 0.01, ***P ≤ 0.001).

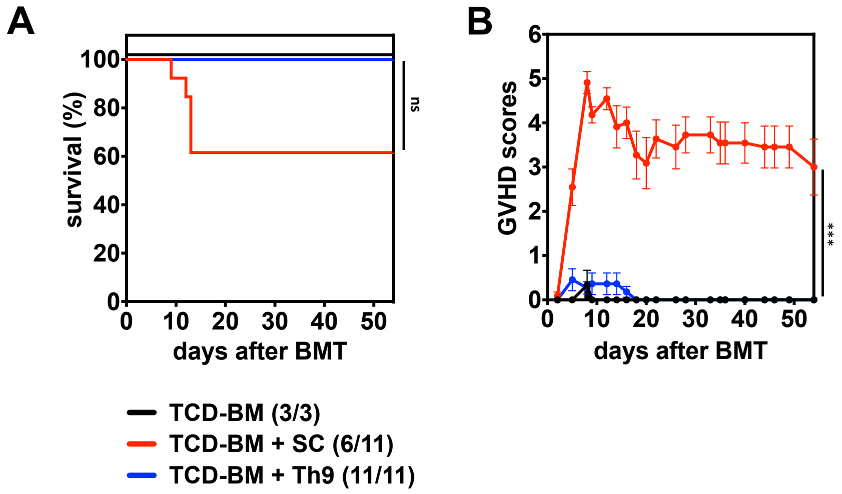

Figure S4. Th9 cells do not induce clinical GVHD in a CD4^{+}-dependent single MHC class II-disparate BMT model.
Lethally irradiated B6.bm12 (I-A^{bm12}) mice were reconstituted with B6-derived (I-A^{b})TCD-BM with or without B6.SJL-derived derived spleen cells (SC)(I-A^{b}) or in vitro-generated Th9 (I-A^{b}) cells. (A) Surviving animals/total animals treated are indicated in brackets and represent data from a single experiment. (B) Error bars of GVHD scores indicate mean ± SEM; ns = not significant; ***P ≤ 0.001).

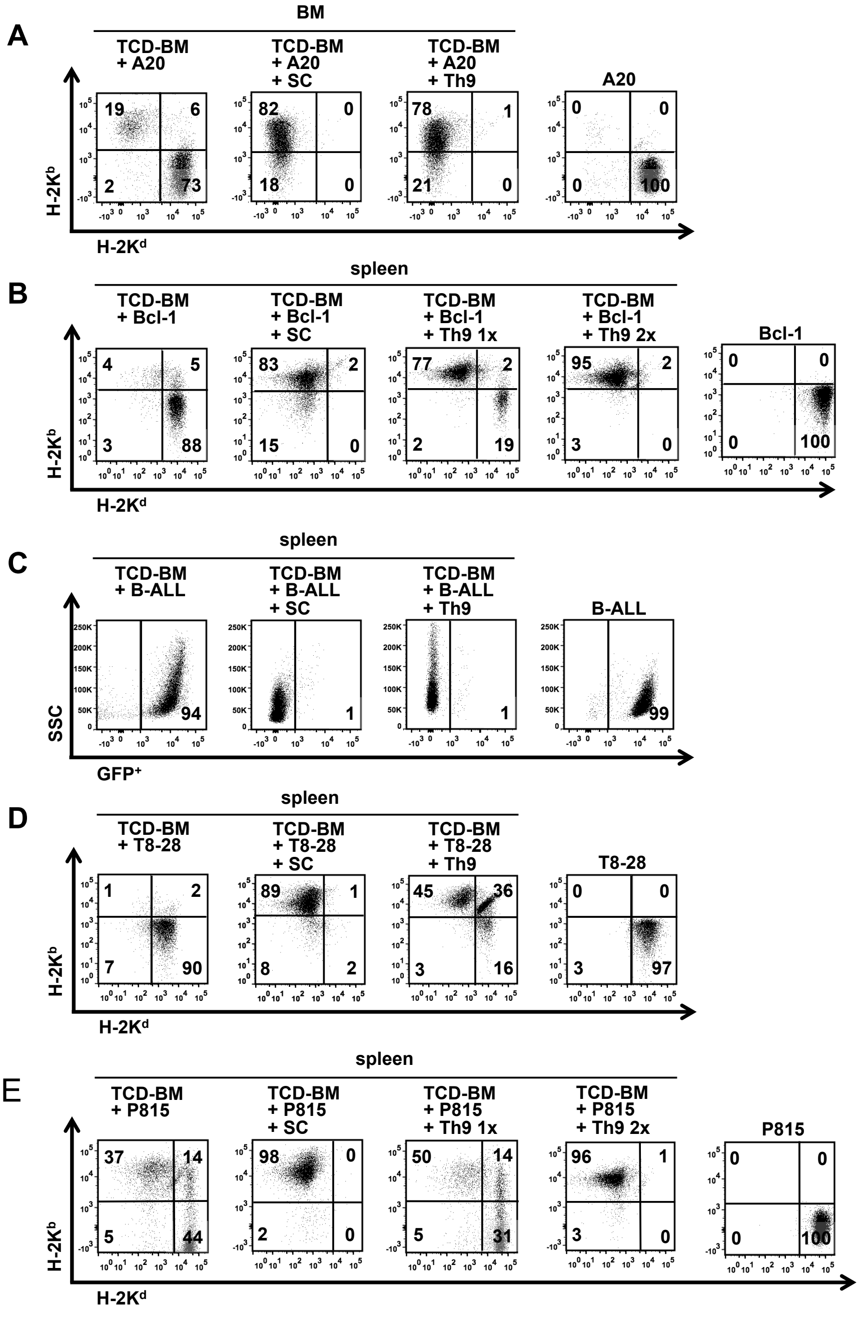

Figure S5. Th9 cells eradicate B cell malignancies.
B6D2F1 mice were reconstituted with B6-derived TCD-BM in the absence or presence of B6-derived SCs or Th9 cells. Mice were co-injected with A20(A), Bcl-1 (B), Bcr-Abl+ B-ALL (C), T8-28 (D) and P815 (E). Presence of tumor cells were defined by expression of H-2K^d (A, B, D, E) or GFP (C). 1 representative staining of one mouse from at least 5 mice analyzed is shown.

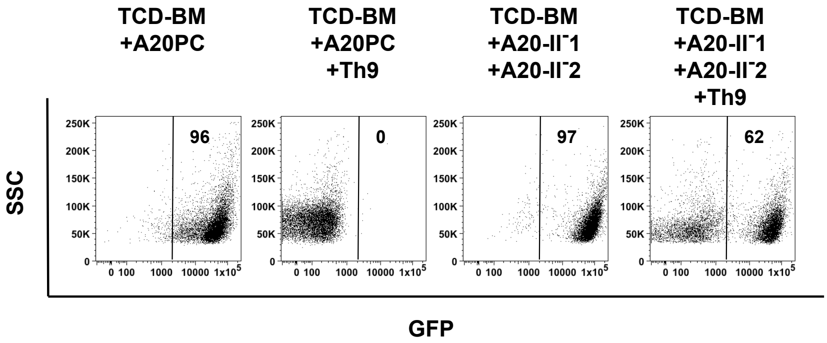

Figure S6. MHC Class II deficient A20 cells form tumors in Th9-reconstituted mice

B6D2F1 mice were transplanted with B6-derived TCD-BM and a 1:1 mixture of GFP^+A20 MHC Cl $II^{-/-}$ cells (A20-II^-1+A20-II^-2) or the GFP^+MHC Cl II^+ A20PC in the absence or presence of Th9 cells. BM cells were analyzed for the presence of tumor cells by GFP expression at the end of the experiment or the day mice were euthanized due to their moribund state. FACS analysis is shown for 1 representative mouse out of at least 5 mice analyzed.
